# A Mixed Model Approach for Estimating the Optimal Food Fortification of Vitamin D: Experiment Based on Mashhad Cohort Study in Iran

**DOI:** 10.34172/aim.2023.82

**Published:** 2023-10-01

**Authors:** Marjan Pourmohamadkhan, Zahra Khorasanchi, Hamideh Ghazizadeh, Atefeh Sedighnia, Behzad Kiani, Omid salemi, Gordon Ferns, Sharareh Rostam Niakan Kalhori, Majid Ghayour-Mobarhan

**Affiliations:** ^1^Department of Health Information Management, School of Allied Medical Sciences, Tehran University of Medical Sciences, Tehran, Iran; ^2^Department of Nutrition, Faculty of Medicine, Mashhad University of Medical Sciences, Mashhad, Iran; ^3^International UNESCO Center for Health-Related Basic Sciences and Human Nutrition, Mashhad University of Medical Sciences, Mashhad, Iran; ^4^Health Technology Incubator Center, Zahedan University of Medical Sciences, Zahedan, Iran; ^5^Department of Medical Informatics, Faculty of Medicine, Mashhad University of Medical Sciences, Mashhad, Iran; ^6^Department of Biology, Science and Research Branch, Islamic Azad University, Tehran, Iran; ^7^Division of Medical Education, Brighton & Sussex Medical School, Falmer, Brighton, Sussex, UK University of Medical Sciences, Mashhad, Iran

**Keywords:** Deficiency, Food fortification, Fortification mode, Vitamin D, Vitamin D food fortification

## Abstract

**Background:**

Vitamin D deficiency is a prevalent problem in worldwide healthcare related to several system disorders. Food fortification as a solution is associated with several challenges including insufficient coverage of the entire population, required degree of fortification, the vehicles used for fortification and potential toxicity. This study aimed to determine the optimal amount of vitamin D for fortification without surpassing the upper intake level (UL) of intake at the 95th percentile of the Iranian population and compare two methods of food fortification.

**Methods:**

This study is aimed to develop a model of two different fortifying approaches related to an available dataset called MASHAD cohort study. The dataset comprised demographic and nutritional data of 9704 Iranian individuals living in the Greater Mashhad region. The first approach was a computational method necessary to implement a range of eight foods and calculate the optimal approach. In the second case, we used the European formula method called ILSI.

**Results:**

To find the appropriate value for fortification, we calculated the consumption of 400 IU and 1000 IU supplements of vitamin D. Three micrograms per 100 g in each food was the optimal output. We also used Flynn and Rasmussen’s formula on our data. Using these methods, we found that 2.1 micrograms per 100 kcal provides the best result. Hence, using the two different approaches, the results appear to be consistent and promising.

**Conclusion:**

One interesting finding was that supplement consumption did not greatly affect the impact of fortification. This observation may support the hypothesis to determine the amount of fortification, and we can ignore the study population’s supplement consumption.

## Introduction

 There are more than 1 billion people with vitamin D deficiency globally.^1^ According to the National Food and Nutrition Surveillance (NFNS) report, over 70% of Iranian adults and children have vitamin D deficiency/insufficiency.^2^

 Vitamin D is an essential nutrient that is principally known for its role in the metabolism of calcium, fertility, and general health. It helps to prevent diseases like cardiovascular disease (CVD), cancer, inflammatory bowel disease (IBD), type 1 diabetes, immunological disorders, and infectious diseases.^3^ The gut’s ability to absorb calcium and phosphorus depends on vitamin D. Lack of vitamin D is a metabolic problem. Bone diseases are a serious problem that can cause pathologic fractures, osteomalacia, and osteoporosis in adults, as well as skeletal deformities, low stature, and delayed growth in children.^4,5^

 The sources of vitamin D include a proper and balanced diet, supplements, and sun exposure.Concerns about excessive sun exposure and skin cancer risk have made the fortification strategy a better option to improve vitamin D dietary intake as an option in the future.^6^ Vitamin D deficiency in Iran is a challenging health issue that requires urgent attention to prevent adverse consequences.^7^ Consumption and fortification of foods is recommended to achieve a satisfactory vitamin D status. The use of supplements is challenging because of the need to provide coverage for the whole population, compliance, cost.^8,9^

 In contrast, studies have indicated that using fortified foodstuff is a more sustainable and cost-effective solution with lower price and higher compliance.^2,10^ The food vehicle should preferably be one of the staple foods in each region to fortify on a big scale with complete population coverage.^2,11^ Moreover, age and season are major factors that need to be taken into account in a fortification program.^12,13^ Initial studies using milk, orange juice, and yogurt drinks as vehicles for tailored fortification prompted the fortification of these products with vitamin D at the request of the market.^14^ In food fortification, it may be observed adverse effects because of excessive in upper limited of vitamin D. Therefore, identifying optimal values of the fortification of vehicles are essential.^15,16^

 Based on the Iranian Food and Drug Administration, fortification is currently optional. The minimum and maximum vitamin D fortification values for dairy products are 30 IU/d and 60 IU/d.^16^

 According to reports, Iranians consume 200 IU of vitamin D per day on average, which is lower than 15 mg/d; 600 IU/d, the U.S. Institute of Medicine’s recommendation for all adults.^16^ Hence, there is a need for vitamin D food fortification according to the developed models.

 Several models have been already developed to determine the greatest quantity of a certain micronutrient that can be added to food without causing toxicity. These models were developed to calculate the total amount of micronutrients added to a specific food or food portion’s 100 kcal.^14,17^ These models were developed to calculate the maximum micronutrients that can be added to a food’s 100 calories with no adverse effect.^18^ Because of the potential adverse effects, it is important to determine safe and efficient methods for food fortification. Different fortification scenarios may be implemented. This paper modeled different scenarios with the fortification of diverse foods such as dairy, butter, and flour. This model presents a mathematical model to identify the optimal fortifying food level and compare Flynn and Rasmussen’s models in the Iranian population in MASHAD Study (2008–2012). We also simulate the effect of a variety of fortified food and coverage, toxicity, and deficiency in our study population.

## Materials and Methods

 Based on the dietary consumption information from the MASHAD study, the data are derived from a cohort of 9704 individuals aged 35–65 years including food frequency questionnaire (FFQ) and 24-hour food records.^17,19^ We chose popular foods that may be used for fortification. These three groups of foods are frequently consumed daily in Iran.^20^ We selected flour, dairy, and butter as vitamin D fortification food vehicles in a simulation model to anticipate the impact of food fortification on vitamin D status. The recommended dietary allowances (RDA) and upper intake level (UL) were used as thresholds based on the previously recommended RI (40 μg /d), and UL (250 μg /d).^14,16^

 We modeled two methods of fortification: the first method was to implement fortification in flour and dairy products, and butter was frequently consumed and was selected for fortification with vitamin D3. We fortified the eight most consumed foods, fortified by vitamin D, in the defined range of fortification.

 Based on Mendes et al^1^, the amount of vitamin D received from fortified foods was calculated, whereas *a*_i_ the amount of consumption by the determined vehicles in our data was calculated. Division by 100 is for obtaining the number of 100 g meals. J to k are variables for fortifying from 1 to 4 microgram applied for all data and were calculated amount simulated received vitamin D to decrease average of vitamin D deficiency in our population. We should emphasize that individual analyses were considered with no supplement use.


(1) 1100 ∑∀j,k,l,m,n,o,p,q=14 ∑i=1nai*j+bi*k+ci*l+di*m+ei*n+fi*o+gi*p+hi*q

In this study, only the values that did not exceed UL in a day were considered acceptable. Minimizing the percentage of people outside the RI-UL range is another strategy.^21^

 The average amount of received vitamin D in our study population is 4 μg/da, whilst our data suggest that adults should receive 10-20 μg/da.^16^ We used these models on MASHAD data and compares them to our simulation based on computational methods.

 The principles of Model’s ILSI and DFVR^22,23^:

 FA (Rasmusen) = (UL – ( CI95 + SI)) /(EI95 * PFF)

 FA (Flyn**)** = (UL - CI95)/ (0·5 * 42 *PFF)

- FA: the safe amount of each nutrient that can be included in a 100 kcal* serving
- UL: tolerable upper intake level
- CI95: current intakes of micronutrients from non-fortified food at the 95th percentile
- SI: supplement intake
- EI95: use of energy at the 95 percentile currently
- PFF: the percentage of marketable foods that can be fortified

The minimum threshold of vitamin D in the IOM standard is 20 IU equivalent to 800 μg/d.^16^

- UL = 100/20 = 5 IOM RDAs;
- CI95Vitamin D = 1/20 = 0.05 IOM RDAs
- MA Vitamin D = UL-CI95 or 5-0.05 = 4.95 IOM RDAs
- The maximum recommended amount of each nutrient that can be added to a 100 kcal serving, taking into account the daily energy intake of adult males at the 95th percentile, which is 4200 kcal per day or 42 portions of 100 kcal. Only 50% of these portions, which is 21 portions, can potentially be fortified with vitamin D
- EI95 index is 18.8 energy intake at the 95th percentile of our population
- PFF is almost 0.11 (8 food of a total of 70 foods)

The model assumes that 50% of foods could be potentially fortified. For the highest energy consumers, the number of 100 kcal food portions that are potentially fortified with vitamin D in the diets would be 21 portions:

 21 * 0.5 = 10.5 food portions of 100 kcal

 Divided by the total number of fortified food portions consumed, MA Vitamin D represents value that may be safely added to each 100-kcal meal. Accordingly, as a percentage of the IOM RDA: 10% of possible fortified foods

- FA(ILSI)Vitamin D = 4.95/2.31 = 2.14 IOM/100 kcal portion (214 % per 100 kcal portion).
- Considering a supplement of 400IU Vitamin D/day
- FA(DFVR) Vitamin D = 4.5/2.31 = 2.10 IOM/100 kcal portion

We calculated the ILSI and DFVR models with supplement and without supplement. 4200 kcal is the amount of calories consumed by the most eater in the studied society. This means consuming 42 meals of 100 g. 4200 kcal is the amount of calories consumed by the most eater in the studied society. This means consuming 42 meals of 100 g.

 Our computational model implements the range of valid fortifications to find the optimal combination of food fortification and exclude each scenario’s vulnerable group. This computational model, which can account for vulnerable groups, is fundamental because these subpopulation groups are at a much higher risk of vitamin D deficiency than their other counterparts.^24^

 We simulated fortifying eight vitamin D vehicles including butter, white bread, whole bread, whole milk, low fat milk, yogurt, cheese, and doogh in the computational model.

## Results

 The results of implementing the Flynn and Rasmussen model for the MASHAD data are shown in Table 1.

**Table 1:** Maximum Safe Amount of Addition Per 100 kcal Food at 95th Percentile Intake

		**% Foods Fortified (of Total Available)**				
Flynn (ILSI)	Supplement	100%	50%	25%	10%	5%
	—	23	47	94	214	471
Rasmussen (DFVR)	400 IU	24	47	95	210	478
	1000 IU				175	

 In our computational model containing eight foods, the optimal result of fortification was obtained at 3 μg/100 g. The output of models in Table 1 is 2.14 μg /100 kcal.

 There were some cases of differences between our simulation and defined models. Defined models are based on the whole population and the average of kcal, 95% CI, and the number of portions of a specific population. We considered all details of dietary intakes for individuals of the data in our calculation, such as consuming each vitamin D vehicle, amount of calories received in a day, gender, age, specific diseases, and disorders. The ILSI and DFVR models recommend a fixed coefficient for every food that could be fortified while we found different values for vehicles.

 In contrast, the Flynn model exclusively considers vitamin D intake from naturally occurring dietary sources as the foundation for predicting the levels of fortification. The Rasmussen model also takes supplemented vitamin D intake into account. These two models use equations to provide fixed values.^25^ It is evident in Table 1 that the result of both equations is the same. Therefore, in our model, we simulated the fortification.

 The result of fortifying food at several percentages is shown in Table 2. 10% fortification equivalents of 8 foods of 70 items.

**Table 2:** Percentage of Meals Fortifiable to 21 Portions of 100 kcal

**% Potentially Vitamin D-Enriched Food Energy**	**Maximum Vitamin D Supply Per 100 kcal Food Portion (RDA=20 μg)**	
	**% IOM RDA**	**μg**
5%	471%	94
10%	214%	43
25%	94%	19
50%	47%	9.5
100%	23%	4.6

RDA, recommended dietary allowances.

 In Table 3, vitamin D intakes in selected European countries are compared with Iran as calculated by the ILSI model.

**Table 3:** Consumptions of Vitamin D from all Sources in Several European Countries Compared with Iran Calculated by the ILSI Model^22^

	**IOM RDA**	**Denmark**	**France**	**Ireland**	**Germany**	**Holland**	**UK**	**Iran**	**Representative Value (Means)**
Vitamin D μg	5	1.8	1.3	1.7	3.4	1.8	2.0	2.1	2.0

RDA, recommended dietary allowances.

 In Table 4, the results of the two methods are compared. Results are related to 8 foods equivalent to 10% of our foods. As a result of consuming 800 g of our vehicles, we received 24 μg in the first method and 28.7 μg in the second.

**Table 4:** Comparison of Fortifying Eight Foods from 70 (10% of Total Foods) Method 1 and Method 2 in 800 g of 8 Vehicles

	**Food**	**Method 1**^a^	**kCal per 100 g**	**Method 2**^b^
1	Whole bread	3	37.5	2.1*0.37
2	White bread	3	37.5	2.1*0.37
3	Whole milk	3	62.5	2.1*0.62
4	Low-fat milk	3	50	2.1*0.5
5	Yogurt	3	66	2.1*0.66
6	Doogh	3	50	2.1*0.5
7	cheese	3	166.6	2.1*1.67
8	butter	3	900	2.1*9
total		24	1370 kcal per 800 g	28.7 μg /800 g

^a^μg/100 g. ^b^μg/800 g.

## Discussion

 We aimed to test and compare two approaches to simulate food fortifications of vitamin D in Iran, using data from the Mashhad stroke and heart atherosclerotic disorder (MASHAD) study. In the first approach, we simulated fortification on eight foods from 70 popular foods. It means that almost 10% of our food to fortifying in the second approach. These two approaches yielded similar results. The relatively small difference between the two methods is due to computational considerations. In Flynn’s method, we use the average caloric intake of the entire population. In contrast, in the second approach, we use booster values for individual participants and emphasize the non-toxicity of even one individual from the population.

 Therefore, the Flynn method led to an upper value for fortifying. However, some models are designed based on supplement consumption,^18,23,26,27^ proving that supplement does not have an essential role in determining the value of fortification.^22^ To determine the optimal safe amounts for dietary supplements and fortified meals, several approaches have been developed.^22,23,26,28^ A few studies suggest that the current vitamin D intake recommendation is underestimated, and should be increased to maintain adequate serum vitamin D concentrations.^29^ Previous studies on food fortification with vitamin D have worked on limited data, whereas the sample size of our study is considerable. Moreover, we compared two approaches to fortifying. The formula method is based on the average consumed kcal, and the possibility of adverse health effects from excessive consumption is low.^17,30^ A comparison of recommended values for fortification in Europe and Iran showed that the average value in Europe is 2.0 μg/100 kcal, while the value obtained in Iran was 2.1 μg/100 kcal because of the low intake of vitamin D sources such as fish and dairy.^12,15^

 Several studies simulated the fortification of vitamin D to predict optimal vehicles and values to fortify in some countries.^13,31,32^ Previous RCTs have evaluated the effects of vitamin D–fortified foods such as bread, orange juice, yogurt drink, cheese, and milk.^33,34^ Sharifan et al investigated the impact of low-fat dairy products with 1500 IU of nano-encapsulated vitamin D3 on cardiometabolic indicators in people with abdominal obesity. Intake of fortified dairy products with nano-encapsulated vitamin D3 was linked to better lipid profiles, glucose homeostasis, and anthropometric indices, especially in people who consumed fortified milk.^35^ Madsen and colleagues examined the effects of increasing the vitamin D intake to the recommended amount by fortifying bread and milk and serum vitamin D levels. This study revealed that fortification of milk and vitamin D-enriched bread increases serum 25(OH)D levels during winter.^31,35^

 A strength of this study is modeling two different approaches for the assessment of fortification. This study also used a statistical method for estimating the optimum level of fortification. These results would seem to suggest that, in general, we may use the fixed formula considering a lower threshold.

 We determined the optimal vitamin D intake in fortified foods using two approaches. As the permitted level for vitamin D fortification is implemented as a general fortification regulation, the model would help Iran’s policy-making. Also, this method could be applied to other micronutrients and minerals. We conclude that fortifying food with vitamin D in proper values would be a safe and efficient solution to reduce the number of individuals with low vitamin D intake.

## Conclusion

 The primary objective of the present investigation was to identify the maximum safe level to help policymakers set optimal amounts for the safe addition of vitamin D for food fortification for adults in Iran. The model was also designed to compare the results of two models for identifying the optimal level of fortification. The findings of this study suggest that 2.1 micrograms per 100 kcal with 10% of potential food. 10% of foods means 8 main foods that can be enriched out of 70 main foods. This new understanding should help to improve prediction of the impact of food fortification on a range of foods to ensure the safety of 95% of the adult population. This study is the first study which simulates several models to anticipate feedback on fortifications. The absence of data about infants and children limited this study. Despite its limitation, the study advances our knowledge of the validation of the proposed model.
